# Pore water pressure responses in silty sediment bed under random wave action

**DOI:** 10.1038/s41598-019-48119-y

**Published:** 2019-08-12

**Authors:** Jianwei Niu, Jishang Xu, Ping Dong, Guangxue Li

**Affiliations:** 1 Key Lab of Submarine Geosciences and Prospecting Techniques, MOE, Qingdao, 266100 China; 2 College of Marine Geosciences, Ocean University of China, Qingdao, 266100 China; 3 School of Engineering, University of Liverpool, Liverpool, L69 3GS UK; 4 Qingdao National Laboratory for Marine Science and Technology, Qingdao, 266100 China

**Keywords:** Engineering, Physical oceanography

## Abstract

We studied pore water pressure responses in silty seabed under random wave action through a series of experiments in a wide wave flume. Unlike previous experiments involving regular waves, we focus on random waves including wind-induced short waves and long waves so as to gain further insights into seabed responses and liquefaction risks posed by random waves. In particular, the study investigated how the secondary long waves that were induced by incident short wave groups affected the seabed responses. The test results revealed that these long waves could cause much larger seabed responses than the short waves (eight times larger in our flume tests). Although they had smaller wave heights than the short waves, the long waves were found to contribute much more significantly to the cumulative pore pressure than previously recognized. The likely reason is that the long waves are disproportionally effective in generating cumulative excess pore pressure, confirming qualitatively some of the earlier theoretical predictions. One of the implications from these research findings is that the existing design methods when applied to random waves could grossly underestimate liquefaction potential in silty sediment bed if either spectrum-based mean wave parameters or significant wave parameters were used.

## Introduction

The pore pressure response of seabed sediment is a key parameter used to assess seabed instability under severe hydrodynamic loading conditions. When a seabed that contains loosely deposited fine sediments is subject to cyclic wave pressure, pore pressure tends to develop in the soil. If the magnitude of pore pressure exceeds the overlying effective soil weight, liquefaction can occur, in which case soil particles will behave like a heavy fluid and the soil bed will lose its structural strength. In rapid depositional environments, such as estuarine delta, weakly consolidated slopes that form as a result of loose sedimentation can become unstable and fail spontaneously because of increased excess pore pressure caused by storm waves. This type of failure can result in sections of buried light pipelines to floating to the surface of the seabed, and heavy objects such as breakwaters, caissons, sea mines sink into the seabed. Many incidents with catastrophic consequences have occurred as a result of wave-induced liquefaction of the seabed or the reduction of the structural strength of seabed sediment^1–4^. Therefore, proper evaluation of seabed instability surrounding marine structures is particularly important for engineers involved in the design of foundations for marine infrastructures.

Ocean surface waves are composed of a large number of random components, including wind-induced short waves and a spectrum of secondary long wave (also called infragravity waves) components through subharmonic interactions and other generation mechanisms. Typical short-wave frequencies are between 0.04 Hz and 1 Hz, whereas long-wave frequencies generally are between 0.004 Hz and 0.04 Hz^5^. When ocean waves propagate over a seabed, pore pressure responses are the combined results of all these wave components. Up until now, little is known about the roles played by those long waves in excess pore water pressure generation compared with short waves.

In the past two decades, many studies have investigated seabed liquefaction under progressive wave action^6–13^. On the basis of laboratory observations and field measurements, two distinct mechanisms for wave-induced pore pressure development and soil response have been identified: oscillatory pore pressure and residual pore pressure. The former is the result of instantaneous upward seepage force under the wave troughs, while the latter is caused by the accumulation of excess pore pressure as the result of soil contraction under cyclic wave loading^14^. Recently, a large amount of research has been conducted on the wave-soil-structure interactions to understand the spatial and temporal behavior of excess pore pressure and the mechanism of seabed instability surrounding coastal structures^15–19^. Despite these efforts, our understanding of how excess pore pressure develops remains rather incomplete as most previous theoretical and experimental studies are limited to regular and periodic waves, whereas real coastal waves are irregular and random. At present, detailed data are lacking on excess pore pressure development and liquefaction potential in random waves, either in the field or in the laboratory.

Among the limited studies on the problem of wave-seabed interactions under random waves, most notable is that of Sumer^11^ who investigated the effect of irregular waves on seabed responses through experimental tests, and suggested that the process of pore pressure buildup in random waves is qualitatively the same as in the regular waves. However, Sassa *et al*.^12^ reported a field observation of the build-up and dissipation of excess pore pressure under storm waves and found that the variation in residual pore pressure was closely related to wave-grouping characteristics in terms of the spectrum. Maeno and Hasegawa^20^ proposed a simple theoretical relationship between the fluctuation of pore-water pressure and wave pressure based on the measurement of wave-induced pore-water pressure at the surface of the seabed.

In addition to laboratory and field studies, theoretical analyses on random wave–seabed interactions can be found in some published studies. On the basis of the dynamic model of Zienkiewicz *et al*.^21^, Wang *et al*.^22^ developed a finite element model to numerically examine the effect of random waves on wave-induced pore pressure and effective stress. Liu and Jeng^23^ established a semi-analytical solution for random wave-induced soil response based on the framework proposed by Jeng^24^. By comparing B-M and JONSWAP spectrum, the influence of random waves on seabed response was investigated. Zhou *et al*.^25^ investigated the effects of random waves on seabed by comparing them with corresponding Stokes waves and Cnoidal waves based on numerical results. All of these theoretical and numerical analyses may reflect the mechanism of random wave–seabed interactions to a certain extent and suggest more liquefaction potential than in corresponding regular waves, but they are limited by the generation of random waves and have not simulated the long waves induced by incident wave groups. The work that is perhaps most relevant to this study is that of Xu and Dong^26^, who investigated the effect of randomness of wave height on seabed liquefaction by adopting an ensemble modeling approach based on a two-layer inviscid fluid flow model. Their modeling results indicated that the random wave-induced liquefaction depth is much larger than that corresponding to regular waves with equivalent wave height. This larger liquefaction depth in random waves has been shown to be due to the fact that the highest waves, rather than average waves, in a wave series tend to dominate the liquefaction extent.

**Figure 1:**
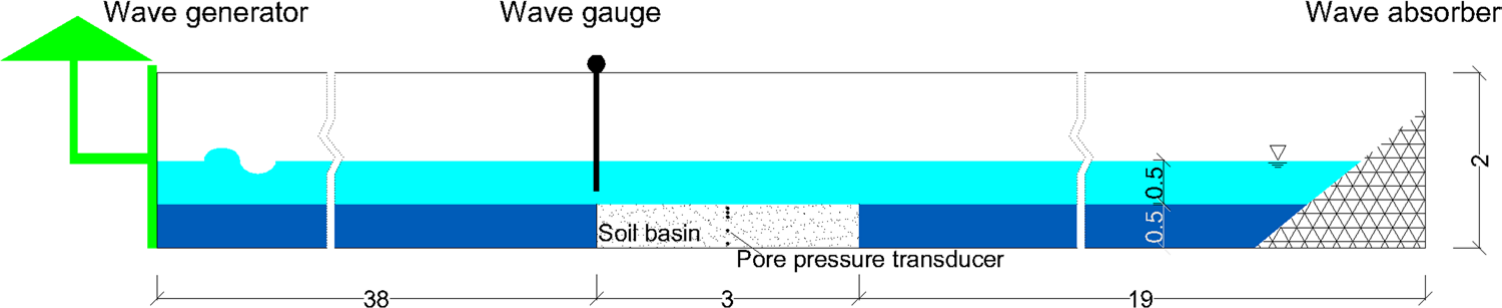
Experiment flume setups in longitudinal sections (unit: m).

To provide quantitative insight into pore pressure responses under random waves, especially long waves, we conducted a unique large-scale flume experiment at Ocean University of China. As shown in Fig. 1, the wave flume was 60 m long, 3.0 m wide, and 2.0 m high. A sediment pit (3.0 m by 3.0 m by 0.5 m) was located 38 m from the wave generator and 15 m from the wave dissipation zone. The sediment pit in the middle of the flume was filled with silt sediment taken from the Yellow River delta, which is known to have strong liquefaction potential, and the same sediment has been used in previous studies involving regular waves^13^. The flume was equipped with a hydraulically driven piston-type wave generator at one end and a 1:3 dissipating beach covered with energy absorbing material at the other end to mitigate the wave reflection. We measured pore pressures at a number of locations in the bed to show how pore pressure develops in the silty bed under the designed random wave sequences. We recorded water-surface elevations with a capacitance wave gauge located at the edge of the sediment pit. The absolute accuracy of this wave gauge was ± 1 mm. Both regular and random waves could be generated by the wave generator with a fully programmable controlled motor.

We used the silt sediment samples from intertidal mudflat in the Yellow River delta as its great liquefaction potential leading by poor drainage. The pore pressure transducers were fixed at different depths of the bed. We carefully prepared the experimental seabed and recorded the changing of soil properties during the consolidation process (the detailed record and the procedure can be seen in the part of Method).

We conducted tests for two series. We generated random wave series using conventional JONSWAP spectra with significant heights from 10.61 cm to 14.39 cm and used regular wave series for comparison. The wave properties are given in Table 1 in which *H*_*m*_ is the average wave height, *H*_*s*_ is the significant wave height, and *T*_*m*_ is the average wave period. The total length of wave action time was 1200 s and included approximately 800 individual waves. The water level was set to 0.5 m. Based on tests results, we discussed the possible mechanisms and engineering implications based on experimental data.

**Table 1:** Test conditions.

Test series	Wave conditions		
	*H*_*m*_ (cm)	*H*_*s*_ (cm)	*T*_*m*_ (s)
1–1^a^	9.47	—	1.50
1–2^a^	13.18	—	1.50
1–3^a^	16.92	—	1.50
2–1^b^	7.06	10.61	1.51
2–2^b^	9.77	14.39	1.56
2–3^b^	9.56	14.14	1.54

*Note: The water depth is* 50 cm.

^a^Regular waves.

^b^Random waves.

## Results

### Characteristics of laboratorial random waves

**Figure 2:**
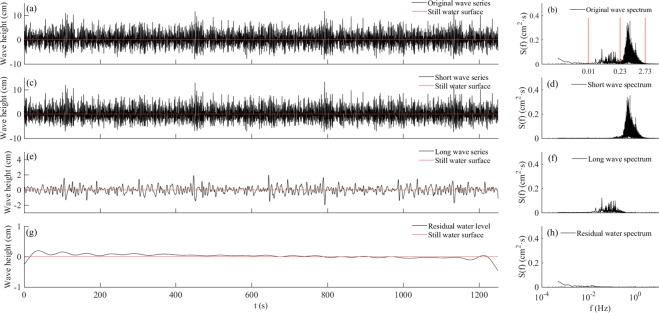
Decomposition of wave series and corresponding Fourier spectra in Test 2-1: (**a**) Original wave series, *s*_*0*_; (**b**) original wave spectrum; (**c**) short wave component *s*_*0s*_; (**d**) short wave spectra; (**e**) long wave component, *s*_*0l*_; (**f**) long wave spectra; (**g**) residual water level, *s*_*0r*_; and (**h**) residual water spectra.

Figure 2 shows the measured time series of water level fluctuations in Test 2-1, in which the random wave was generated using JONSWAP spectrum with a significant wave height, *H*_*m*_ = 10.49 cm; mean wave height, *H*_*m*_ = 6.94 cm; and peak frequency, *fp* = 0.533 Hz. Figure 2(a,b) show that original random waves s_0_ contained various wave components that were distributed along different frequency bands. By filtering, short wave *s*_0*s*_, long wave *s*_*0l*_, and residual water level *s*_*0r*_ were separated (Fig. 2(c,e,g)); the corresponding Fourier spectra are shown in Fig. 2(d,f,h). As the figures show, *s*_*0s*_ was distributed between 0.23 Hz and 2.75 Hz, and *s*_*0l*_ was distributed between 0.01 Hz and 0.23 Hz.

**Figure 3:**
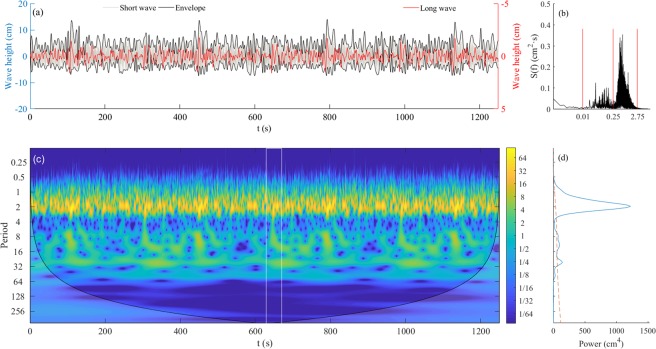
Wave internal structure and energy distribution of random wave in Test 2-1: (**a**) left: wave height of short waves *s*_*0s*_ and their envelope, right: long wave series s_*0l*_; (**b**) Fourier spectrum of random wave in Test 2-1; (**c**) wavelet power spectra of random wave in Test 2-1; and (**d**) global wavelet power spectra of random wave. (The red line indicates significance levels at 95%).

Figure 3(a) shows the short-wave group enveloped and bound long waves *s*_*0l*_ in Test 2-1 to reveal their phase correlation. The phase difference between the wave groups and the bound long waves was 180°, so the right y-axis of *s*_*0l*_ in Fig. 3(a) is inversed to provide a direct comparison. The phase correlation shows that bound long waves were the dominant components in the laboratorial long waves.

Figure 3(b) shows the wavelet spectrum of *s*_*0*_, which reflected the energy distribution on temporal and spatial scales. The global wavelet power spectrum (Fig. 3(c)) provided a comparison of power spectral intensity between the low-frequency (LF) and high-frequency (HF) band. From the wavelet spectrum of these waves, it is evident that some LF energy across a large frequency range (e.g., the white box in Fig. 3(b)), most likely caused by wave breaking in which short waves transfer some of its energy to long waves. From the energy spectrum, it is evident that the energy of *s*_*0l*_ was much lower than *s*_*0s*_, and wave breaking could generate long waves.

### Pore pressure response under random waves

**Figure 4:**
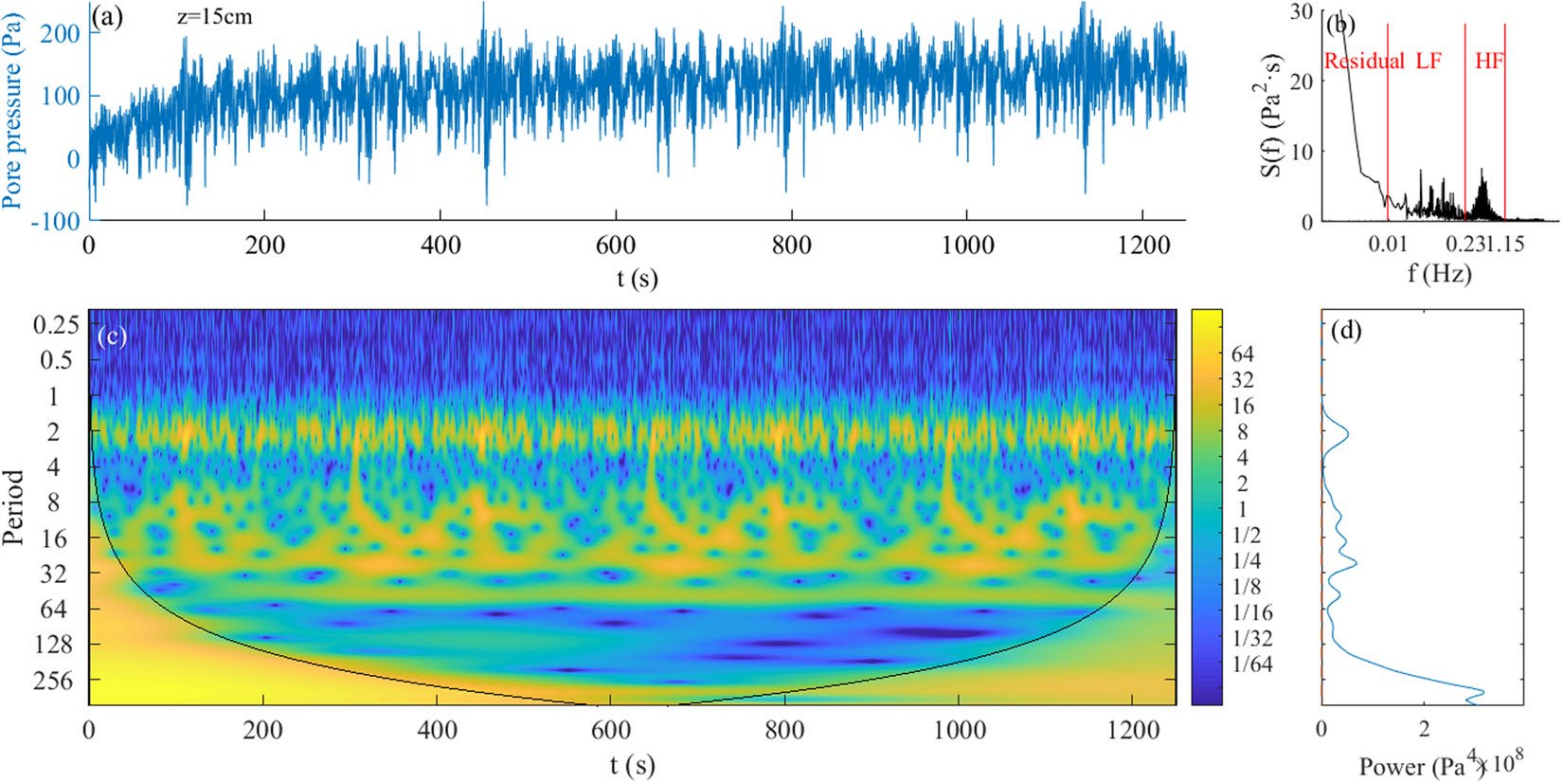
Pore pressure series and its energy distribution in Test 2-1: (**a**) pore pressure at 15 cm depth of bed in Test 2-1; (**b**) pore pressure Fourier spectrum with different frequency bands; (**c**) pore pressure wavelet power spectrum; and (**d**) pore pressure global wavelet power spectrum.

The pore water pressure measurements showed both a rapid response and a gradually changing trend, which corresponded to oscillating and residual pore pressures, respectively. Seabed responses were more complex because of complex hydrodynamics under random waves. Pore pressure responses to secondary waves and incident waves were distinct. Taking the pore pressure at a depth of 15 cm from Test 2-1 as an example, the time series, Fourier spectrum, wavelet power spectrum, and corresponding global wavelet spectrum of the pore pressure are shown in Fig. 4.

With continuous wave action, the accumulation of pore pressure lasted for a much longer time, resulting in much larger residual pore pressure (Fig. 4(a)). Moreover, the pore pressure continued to accumulate after it reached quickly to approach the initial peak, although the rate of accumulation was much smaller than the rate during the initial period. This is different from that seen in regular wave experiments in that pore pressure remained more or less unchanged after reaching the peak. This variation trend also occurred at other depths.

According to the Fourier spectrum, the energy distribution of pore pressure (Fig. 4(b)) was similar to that of the random waves (Fig. 3(b))—that is, *p*_*0s*_ and *p*_*0l*_ oscillated at the same frequency as *s*_*0s*_ and *s*_*0l*_. Note that the residual pore pressure *p*_*0r*_ was cumulative and increased with wave action. Therefore, its spectrum form would be shown as the monotone increasing along with the decrease in frequency. Note that the frequency range of *p*_*0s*_ was much smaller than *s*_*0s*_ (Figs 2(b), 4(b)) as a proportion of short waves could not reach the bed and thus do not contribute to the pore pressure generation. More detail can be seen from the pore pressure wavelet spectrum in Fig. 4(c), which shows that the energy of *p*_*0s*_ is suddenly reduced markedly at T = 1.3 s, whereas *s*_*0s*_ in Fig. 3(c) is not.

### Effect of long waves on pore pressure responses

**Figure 5:**
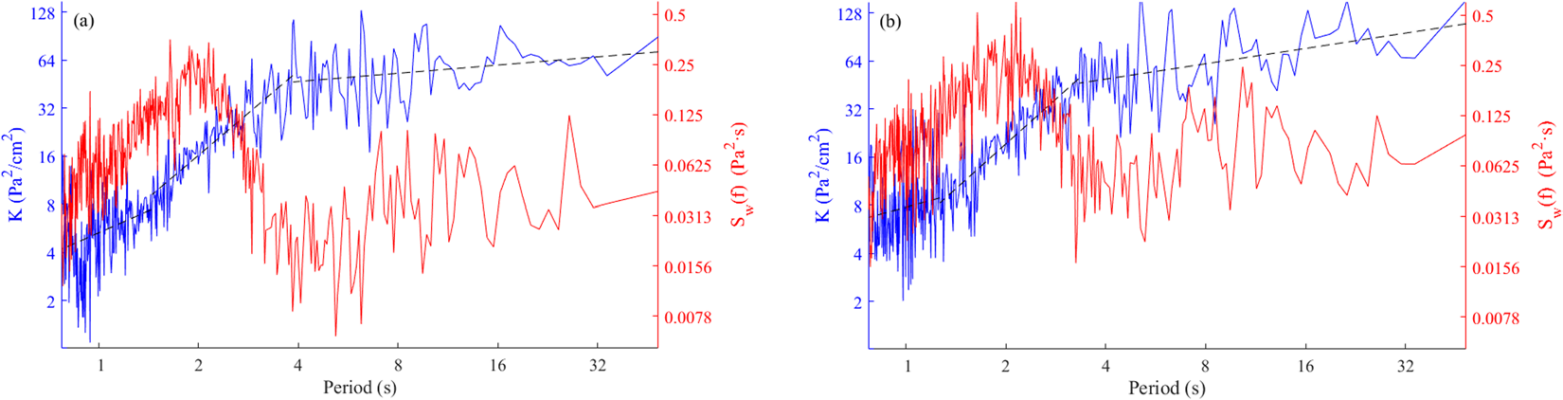
Comparison of the intensity of pore pressure to waves t different frequencies: (**a**) Test 2-1, random waves, *H*_*s*_ = 10.61 cm, *T*_*m*_ = 1.51 s; and (**b**) Test 2–2, random waves, *H*_*s*_ = 14.39 cm, *T*_*m*_ = 1.56 s.

We introduced a parameter *K* to study the intensity of seabed response to different wave periods. The greater *K* indicated a stronger pore pressure response under the corresponding wave period. We defined *K* by the ratio of *S*_*w*_*(f)* and *S*_*p*_*(f)*, where *S*_*w*_*(f)* and *S*_*p*_*(f)* were the Fourier spectrum of individual waves and the corresponding pore pressure response, respectively. The index of *K* in different random wave tests is shown in Fig. 5. The figure shows that the changes of *K* have three major phases with the wave period increasing: (a) initial slow growth phase, (b) rapid growth phase, and (c) very slow growth phase. These three different phases were controlled by the wave period, and the oscillation amplitude of the ratio was controlled by wave height at same frequency. The value of *K* between the two slow growth phases showed that pore pressure responses under long waves were nearly eight times greater than short waves and the relationship was highly similar in Test 2-1 (*H*_*s*_ = 10 cm) and Test 2-2 (*H*_*s*_ = 14 cm) (Fig. 5(a,b)). By comparing the wave spectra, we observed that the change trends of *K* were virtually unaffected by the wave energy distribution, which meant that the wave periods were the key factors for *K*. Furthermore, we inferred that the various wave components (with different wave period) would make different contributions for seabed responses and the changing of water environment would strongly affect the liquefaction of seabed.

### Comparison of random- and regular-wave induced pore pressure

**Figure 6:**
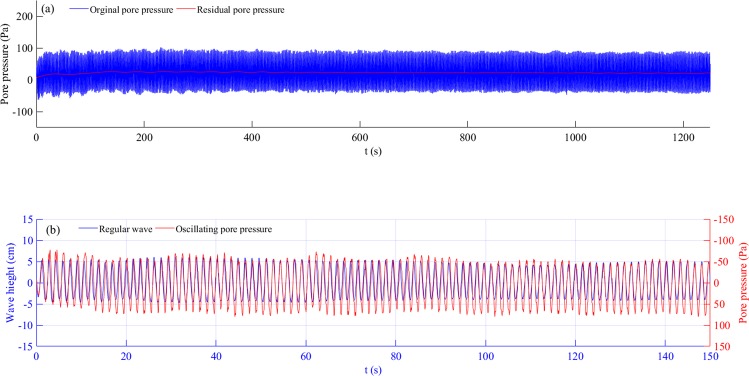
Pore pressure responses under regular waves at depth of 15 cm; regular wave, Test 1–1.

**Figure 7:**
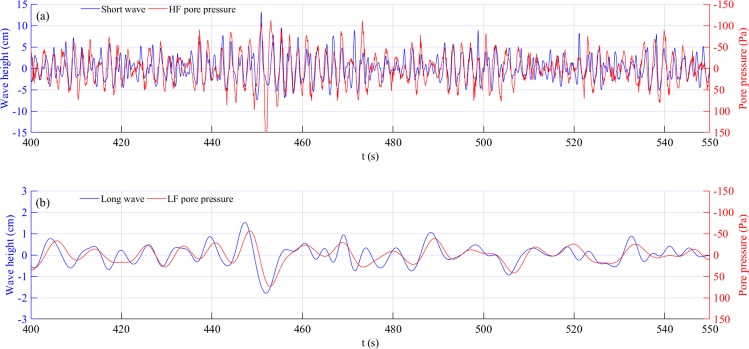
The relationship between different wave components and corresponding pore pressure responses during random waves: (**a**) short wave and HF pore pressure, and (**b**) long wave and LF pore pressure.

The spectrum analyses showed that in random waves, the long waves caused stronger pore pressure responses, although their heights were smaller than short waves, which meant that the traditional tests using regular waves could not reflect fully the actual process of pore pressure development and seabed liquefaction. To evaluate whether the same individual wave that had the same wave height and period would cause equal pore pressure response both in the random waves or regular waves, we conducted comparative experiments. Taking the first group as an example, regular Test 1–1 (*H*_*m*_ = 9.47 cm, *T*_*m*_ = 1.5 s) and random Test 2-1 (*H*_*m*_ = 7.06 cm, *H*_*s*_ = 10.61 cm, *T*_*m*_ = 1.51 s), Figs 6, 7 provide the detail of the wave series and their pore pressure responses. As shown in Fig. 6(a), residual pore pressure clearly accumulated and then remained unchanged under regular waves, which suggested that the regular waves generated a single frequency of pore pressure responses with a nearly identical intensity (Fig. 6(b)). Figure 7 provides the pore pressure responses under short waves and long waves during random wave action. It is evident that the smaller heights of the long waves caused significantly larger pore pressure responses. Note that the pore pressure response caused by the individual regular wave was equal to that caused by the identical short-wave components within the random waves, such as t = 417 s, 495 s (Fig. 7(b)). This suggested that there were no notable differences on pore pressure responses under the same individual wave component between the regular and random waves. Long waves generated by short wave groups or short wave breaking, however, had a significant impact on seabed response, further resulting in more intense responses than regular waves.

**Figure 8:**
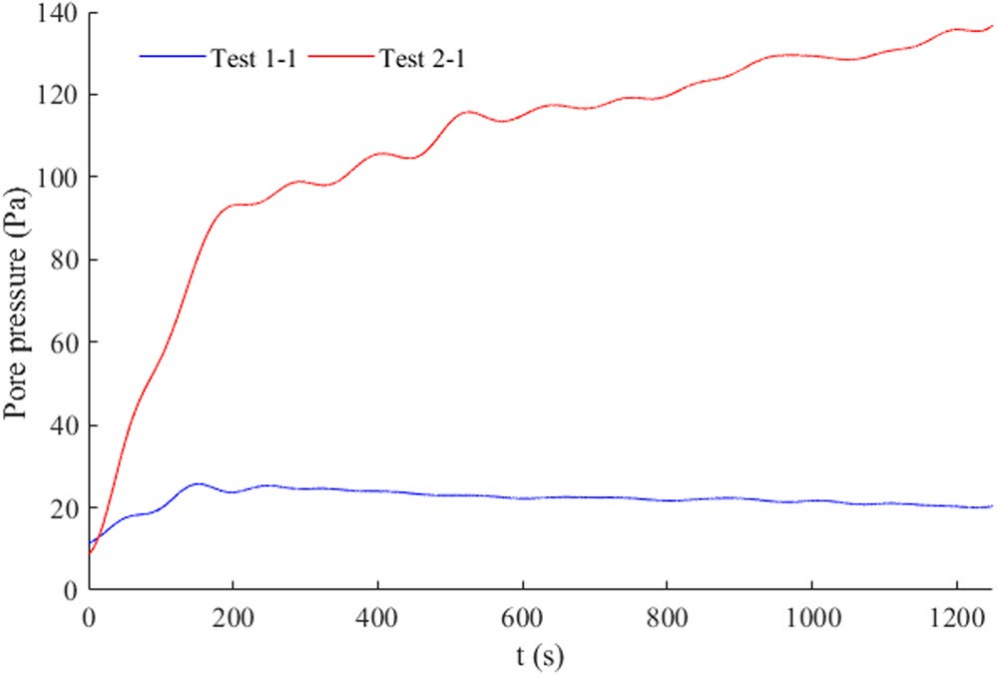
Residual pore pressure development at depth of 15 cm: (a) regular waves, Test 1–1; and (b) random waves, Test 2-1.

Residual pore pressure development is shown in Fig. 8. For regular waves (Test 1–1), the excess pore pressure at 15 cm reached its peak and then reduced slowly, which was likely caused by the discharge of pore water. For the comparable random waves (Test 2-1), however, the excess pore pressure continued to accumulate, although the rate of accumulation was much smaller than that in the initial period. Thus, the residual pore pressure under random waves was much larger than the random waves, which implied that random waves had a much greater liquefaction potential.

## Discussions

The experimental results have shown clearly that the pore pressure responded strongly to random waves, especially to long waves induced by the presence of groups in incident short waves. Unlike ocean waves that combine various generation mechanisms of long waves, in these tests, the random waves are limited only to bound long waves and free bound waves, which resulted from the breaking of short waves. Another limitation was that the wave maker could generate only a finite frequency band, and the effects of longer waves with even lower frequencies could not be considered. In the following sections, we discuss the mechanism for pore pressure development and seabed liquefaction under random waves based on our laboratory results.

**Figure 9:**
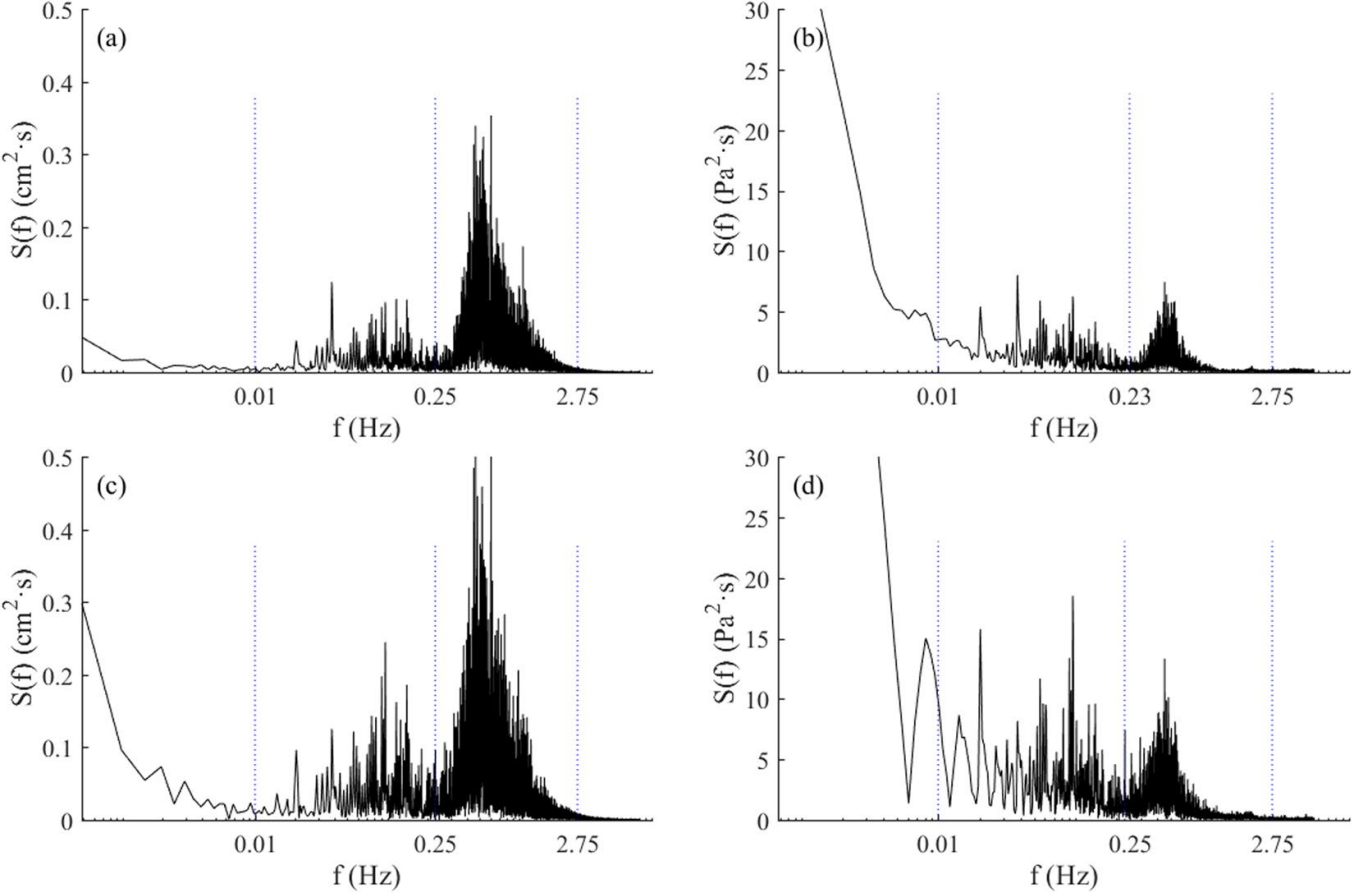
Wave and pore pressure Fourier spectra under different random waves: (**a**) wave spectrum in Test 2-1 and (**b**) pore pressure spectrum in Test 2-1 in 15 cm depth of bed; (**c**) wave spectrum in Test 2–2; and (**d**) pore pressure spectrum in Test 2–2 in 15 cm depth of bed.

First, frequency distribution of random waves is crucial for seabed responses because a significant proportion of high frequency waves are too short to reach the seabed, even when they may have a larger wave height. This was demonstrated clearly by the pore pressure spectra in Test 2-1 (b) and Test 2–2 (d) (Fig. 9) in which the spectral intensity suddenly reduced at the same frequency, although wave height increased significantly from about 10 cm to 14 cm. Second, in these tests, seabed pore pressure responses under long waves were almost eight-fold greater than that under short waves with equivalent wave height. Although this estimate may have certain deviations compared with ocean conditions, it still implied that the conventional assessment method that uses significant wave height and the square root of wave period of random as the wave parameters could vastly underestimate the effect of long waves on seabed responses. This underestimation is especially notable in ocean waves that have extensive long waves accompanied by incident groups of short waves with more complex generation mechanisms. Also note that residual pore pressures under random waves were much larger than the corresponding regular waves (Fig. 8), which likely could be attributed to the effect of the long waves.

Although the experiments are performed for a flat silty sediment bed, some of the finding could also have implications for the general problem of wave-soil-structure interactions. The greater liquefaction potential under random waves is expected to be applicable to structures affected by secondary long waves, which is consistent with the conclusion proposed by Zhu *et al*.^15^. However, the existence of a structure within the seabed may strongly affect the wave field and the distribution of pore pressure and the liquefaction potential surrounding the structure as shown by Jeng^24^, for the case of a breakwater. In addition, the natural frequency of some coastal structures can be that of the short waves (e g., the first excitation frequency of a modern offshore wind turbine was usually in the 0.17–0.33 Hz^27^). Therefore, the resonance of the short wave-soil-structure would also affect the liquefaction potential and stability of offshore structures. The relative contributions to liquefaction potential by the secondary long waves and short waves when structures are present still requires further research through experimental studies and numerical simulations.

Because this phenomenon was only observed in the present laboratory experiments, further field research is essential as naturally occurring waves are much more complex than those in the laboratory. The secondary long wave is extremely important in near-shore seabeds, whereas swell wave components (also with a long wavelength) could also make significant contributions to soil liquefaction in deeper water, such as in an inner-shelf environment.

## Conclusions

Following are our conclusions:

1. Secondary long waves induced by the presence of groups of incident short wave are an important part of random waves and may be responsible for much of the large seabed responses, even though their wave heights are much smaller than corresponding short waves.
2. The residual pore pressure generated by random waves can be much larger than that by regular waves with equivalent mean wave height and period, as the low frequency waves can make a significant contribution to the residual pressure.
3. In assessing the potential of seabed liquefaction and instability it is necessary to consider full random waves instead of using characteristic regular waves with spectrum-based mean wave parameters.

## Methods

### Experimental setup and procedure

We placed the test soil in the sediment pit, which had impermeable concrete walls. Before filling up the flume, we placed a steel frame in the pit on which a number of pore pressure gauges were fixed at depths of 5, 10, 15, 30, and 45 cm. One group of pore pressure gauges was placed at the center of the pit, and another group was located at 0.5 m downstream. The pore pressure gauges were supplied by Nanjing Institute of Hydraulics; these gauges have been used successfully in a number of previous experiments^13^. This arrangement of pressure gauges is unique in terms of the use of a 3 m wide flume, whereas all previous experiments have been conducted using flumes that were much narrower (typically 0.5 m). The wide flume used in this experiment ensured that the pressure gauges were sufficiently far away from the flume walls, and thus, reduced their effects on the behavior of the soil sample and the fluid flow.

### Preparation of test sediments

**Figure 10:**
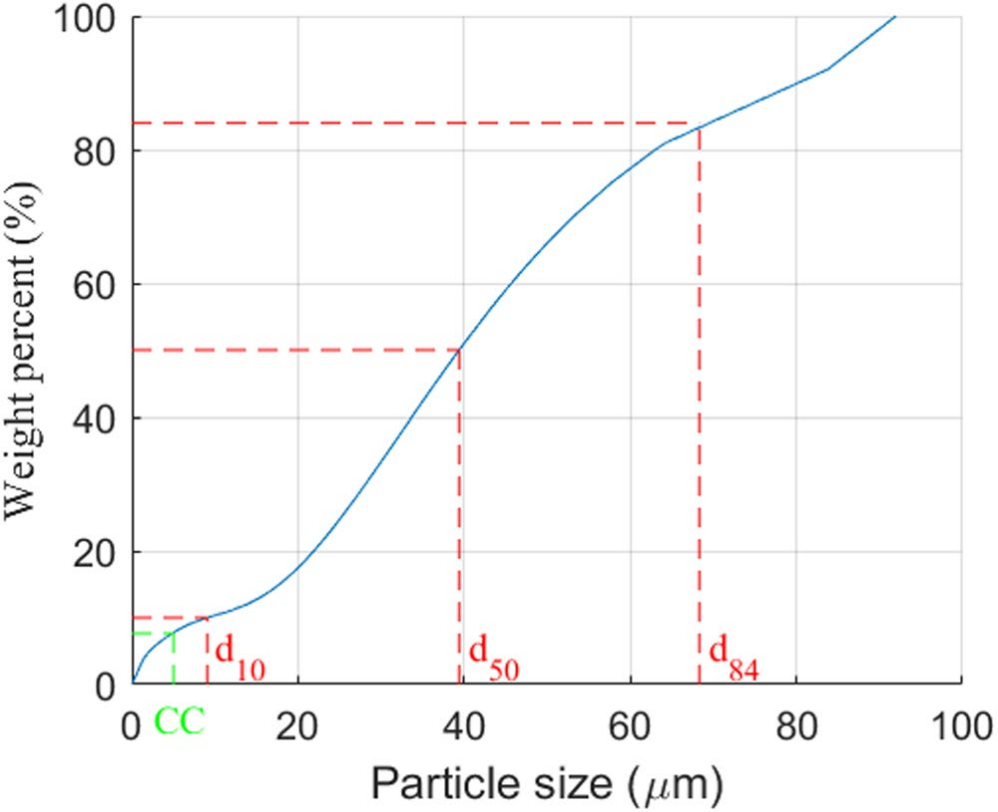
Particle-size distribution of the test sediments.

We collected the silt sediment samples from the intertidal mudflat in the Yellow River delta. The most significant detrital minerals found in the sediment samples were quartz, feldspar, calcite, and dolomite, which accounted for 75.8% of the total mineral composition. The clay minerals were illite, chlorite, kaolinite, and montmorillonite, which accounted for the remainder (24.2%) of the total mineral composition^28^. The grain size accumulation curve is shown in Fig. 10.

We air-dried the silt sediment brought into the laboratory and thoroughly stirred the sediment to avoid agglomeration and ensure homogeneity. The use of remolded soil ensured the repeatability and comparability of the experiments. We then thoroughly mixed the sediment with water in a large mixing tank before placing it into the sediment pit. We repeated the same procedure several times until the sediment pit was full. Some excessive slurry volume was kept above the sediment pit to ensure the presence of adequate silt deposits within the pit after the initial consolidation. The basic soil parameters are listed in Table 2.

**Table 2:** Properties of test soil.

*d*_50_ (mm)	*CC* (%)	*SD*	*φ* (°)	*k*_0_	*s*
0.0394	7.65	1.733	25.4	0.571	2.71

In Table 2, *d*_50_ is the median diameter of the test soil, *CC* is the clay content, *SD* is geometric standard deviation of soil particle, and *k*_0_ is the lateral hydrostatic pressure coefficient representing the ratio of horizontal and vertical effective stresses^29^ and is calculated by *k*_0_ = *1* − *sinφ*, where *φ* is friction angle and can be obtained by the standard geotechnical tests; *s = ϒ*_*s*_/*ϒ*_*w*_ is the density ratio, where *ϒ*_*s*_ and *ϒ*_*w*_ are specific weights of soil and water, respectively.

**Figure 11:**
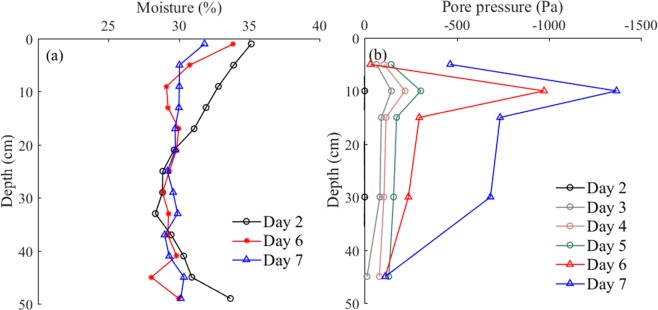
Changes in (**a**) moisture content and (**b**) pore pressure during the consolidation process.

To ensure that the density of the sediment in the pit was similar to that found in the field, the newly placed sediment was allowed to consolidate under its own weight for 5 days. We then filled the flume to the required water level and left the soil for another 5 days. During the consolidation period, some pore water and all air bubbles were expelled, causing reduced porosity and increased soil strength, and the property of soil became more uniform, as shown in Fig. 11(a). It also was evident that the initially placed soil had a relatively large water content, particularly with higher water content in the upper and lower layers than that in the middle layer. As time passed, the sample soil became more uniform throughout the soil pit depth, although it still was not perfectly homogeneous. Figure 11(b) shows the change in pore water pressure during the soil-bed consolidation period, which reflected the drainage speed at different burial depths. It also was evident that the dissipation rate of the pore pressure had obvious differences in vertical during the consolidation process, and this rate increased remarkably after loading with water. The fastest-changing part of the bed was at a depth of 10 cm.

Table 3 lists the properties of the soil sample before and after the consolidation period. The bulk density *ρ* before and after the experiments was 1.86 g/cm^3^ and 1.92 g/cm^3^, respectively; water content *ω* was 38% and 30%, respectively; the corresponding void ratio *e* decreased from 1.000 to 0.835. The degree of saturation is indicated by *Sr*. We determined relative density using the maximum dry density *ρ*_*dmax*_ and minimum dry density *ρ*_*dmin*_. According to the measurements, *ρ*_*dmax*_ = 1.622 g/cm^3^ and *ρ*_*dmin*_ = 1.101 g/cm^3^. The corresponding void ratios *e*_max_ and *e*_*min*_ were 1.1445 and 0.671, respectively. The relative density is calculated according to Eq. (1):

1 $$Dr=\frac{{e}_{\max }-e}{{e}_{\max }-{e}_{\min }},$$

which gives values of 0.36 and 0.78, respectively.

**Table 3:** Mechanical properties of soil in the sediment pit.

Soil type	*ρ*(g/cm^3^)	*ω*(%)	*e*	*Sr*	*ρ*_*sat*_ (g/cm^3^)	*γ*′ (kN/m^3^)
Preconsolidation	1.86	38	1.000	1.012	1.850	8.504
Consolidation	1.92	30	0.835	0.970	1.932	9.319

In this experiment, the averaged *d*_50_ of the test soil sample as 0.036 mm, which was extremely close to the upper limit of medium silt (0.031 mm) on the Wentworth scale^29^. The soil permeability was not directly measured but could be estimated from the previous work using soil taken from the same site (Jia *et al*. 2011b) as ranging from 10^−6^ to 10^−5^.

### Test procedure

**Figure 12:**
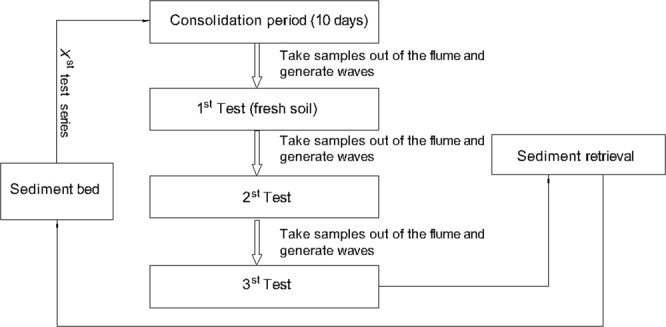
Test sequence for each test series.

Figure 12 shows the test procedure in which *X* represents the *X*_*th*_ test series. Each test series includes three individual tests with progressively increasing wave loads.

We prepared the test and test procedures as follows:

- Clean the flume and sediment pit thoroughly.
- Install pore pressure gauges and place wave-filled bloom to protect the pressure sensors.
- Mix prepared sediment and water thoroughly in a mixing tank, place the soil in the pit, and loosen the soil.
- Leave the sediment in the pit for 12 hours, and after this initial consolidation, level the surface and leave the sediment for 5 days.
- Fill the flume gradually to 50 cm and leave the flume to consolidate for an additional 5 days.
- Install wave gauges and test all instrumentation.
- Take a set of sediment samples, switch on the waves, and continue sampling until the time allocated for the test condition is terminated.
- Switch off the waves, take another set of sediment samples, and repeat three times.
- Empty the flume slowly and clean the flume and sediment pit.
- Check all instruments and prepare for the next test series.
- Take sediment samples for lab analysis.

### Data analysis

Long waves induced by the presence of groups in incident short waves are the most common type of infragravity waves. Bertain *et al*.^5^ summarized the three types of generation mechanism: bound waves, moving breakpoint, and bore merging. A now-recognized consensus is that bound long waves are secondary waves forced by incident short-wave groups with a similar frequency to the wave group. Similar to natural ocean waves, random waves in a flume laboratory contained different wave components and breaking waves (which represented extreme wave-breaking conditions). The wave maker created wave groups with a certain spectrum, and some large waves may have broken during this process. Using the same generation mechanisms as natural ocean waves, our flume laboratory generated both bound long waves and free long waves.

We analyzed the observed data as follows: (a) we filtered the wave data and pore pressure data using a wavelet multiresolution analysis method, (b) we used wavelet and Fourier spectrum to show the energy distribution of data for time and frequency, and (c) we defined the index *K* to describe the seabed response under different wave groups. We did not provide a detailed description of wavelet methodology in this paper, because it has been used widely for wave separation and was not the emphasis of this study (see Różyński,^30,31^). *K* is calculated as follows:

2 $$K=\frac{{S}_{p}(f)}{{S}_{w}(f)},$$

where *S*_*w*_*(f)* and *S*_*p*_*(f)* are the Fourier spectrum of the individual wave and its corresponding pore pressure response, and *K* reflects the intensity of seabed response; the greater *K* is, the stronger the pore pressure response under the corresponding wave.

We divided the observed data of water fluctuation *s*_0_ into the short-wave component *s*_0*s*_, the long-wave component *s*_0*l*_, and the residual water level *s*_*0r*_. The data *s*_*0r*_ had a very large time-scale residual water-level change. Similarly, we divided the data of pore pressure *p*_*0*_ into short-period component *p*_*0s*_, the long-period component *p*_*0l*_, and the residual pore pressure *p*_*0r*_. The short wave *s*_*0s*_ in the present test was forced by wave paddle, and the frequency was distributed across a certain bandwidth. The long waves in the tests had longer periods than the short waves, that is, they were longer than the wave-maker periods.

## Data Availability

The datasets generated and analyzed during the current study are available from the corresponding author on reasonable request.
