# Author Correction: Pore water pressure responses in silty sediment bed under random wave action

**DOI:** 10.1038/s41598-019-53338-4

**Published:** 2019-11-13

**Authors:** Jianwei Niu, Jishang Xu, Ping Dong, Guangxue Li

**Affiliations:** 1 Key Lab of Submarine Geosciences and Prospecting Techniques, MOE, Qingdao, 266100 China; 2 College of Marine Geosciences, Ocean University of China, Qingdao, 266100 China; 3 School of Engineering, University of Liverpool, Liverpool, L69 3GS UK; 4 Qingdao National Laboratory for Marine Science and Technology, Qingdao, 266100 China

Correction to: *Scientific Reports* 10.1038/s41598-019-48119-y, published online 12 August 2019

In the original version of this Article, Affiliations 2 and 4 were not listed in the correct order. The correct affiliations are listed below:

Affiliation 2:

College of Marine Geosciences, Ocean University of China, Qingdao, 266100, China

Affiliation 4:

Qingdao National Laboratory for Marine Science and Technology, Qingdao, 266100, China

This error has now been corrected in the PDF and HTML versions of the Article.

